# Mini Nutritional Assessment (MNA) as a Reliable Tool for Nutritional Assessment of Hemodialysis Patients: A Single-Center Observation

**DOI:** 10.7759/cureus.21024

**Published:** 2022-01-07

**Authors:** Kiran Nasir, Sajid Sultan, Ruqaya Qureshi, Murtaza Dhrolia, Aasim Ahmad

**Affiliations:** 1 Nephrology, The Kidney Centre Post Graduate Training Institute, Karachi, PAK

**Keywords:** maintenance hemodialysis, end-stage renal disease (esrd), chronic kidney disease (ckd), chronic kidney disease (ckd), nutrition status, mini nutritional assessment

## Abstract

Objective

In this study, we evaluated mini nutritional assessment (MNA) as a tool for the assessment of the nutritional status of end-stage renal disease (ESRD) patients on maintenance hemodialysis (MHD).

Methods

This prospective cross-sectional study was done from February 2021 till August 2021 on ESRD patients on MHD at our center. Nutritional status was assessed by using MNA score which evaluates four different aspects: anthropometric measures (body mass index [BMI], weight loss, mid-arm and mid-calf circumferences); general assessment (lifestyle, medications, mobility, and signs of depression); short dietary assessment (number of meals, food, and fluid intake) and subjective assessment (self-perception of food and nutrition).

Results

Out of 195 study subjects, 127 (65.1%) were males and 68 (34.9%) were females. Most women were stay-at-home mothers (57, 29.2% overall and 83.8% among all women), while most men owned their own businesses (44, 22.6% overall). The mean age was 51.2±14 years and the mean duration of hemodialysis was 4.6±4.1 years. Most of our patients belonged to the middle socioeconomic group (110, 56.6%). By using MNA, we found that most are at risk of developing malnutrition (112, 57.4%); however, only 9 (4.6%) patients are malnourished. In our study group, most malnourished patients belonged to the age group of >65 years (5, 56.6%). BMI was found to be significantly associated with MNA (p <0.001).

Conclusion

MNA is an easy and reliable bedside tool that can be used in ESRD patients on MHD for nutritional assessment. This is helpful in nutritional planning and the prevention of malnutrition.

## Introduction

Chronic kidney disease (CKD) is a major public health problem, affecting over 109.9 million people from high-income countries (48.3 million men and 61.7 million women) and 387.5 million from lower-middle-income countries (177.4 million men and 210.1 million women) [1]. With the increasing prevalence of CKD, it is estimated that more individuals will require renal replacement therapies (dialysis or kidney transplant) for end-stage renal disease (ESRD).

Protein energy wasting (PEW) is a frequent finding in patients on maintenance hemodialysis (MHD) with a global prevalence of 28-54% [2], associated with high morbidity and mortality [3,4]. It adversely affects patients' quality of life (QOL) [5,6]. Multiple factors contribute to the development of PEW in MHD patients including losses of amino acids and nutrients, dialysis-induced muscle catabolism, increased energy expenditure, resistance to anabolic hormones, ineffective correction of metabolic acidosis, inadequate dialysis, poor appetite, taste alterations, suboptimal dietary intake, insulin resistance, psychological factors, decreased functional capacity, depression and lack of social support [7].

Nutritional assessment is an important task for providing proper dietary advice to MHD patients. Different methods of nutritional assessment are used include anthropometric measurements (body mass index [BMI], triceps skin fold thickness [TSFT], mid-arm muscle circumference [MAMC] and hand grip strength [HGS]), bio-impedance, subjective global assessment (SGA) and mini nutritional assessment score (MNA) [8,9]. MNA is a self-reported questionnaire-based validated tool for assessing malnutrition, initially used in elderly patients [10]. In a few studies, it was used for nutritional assessment in elderly MHD patients and was found to be reliable [11,12]. Previously we did a study on nutritional assessment using bedside anthropometric measurements [13].

Despite being an important part of management of ESRD patients on MHD, nutritional status is a neglected area, which need proper assessment. Prevention and timely management of malnutrition should be a part of treatment regimen of nephrologist and renal nutritionist. The purpose of this study is to evaluate effectiveness of MNA as nutritional assessment tool in MHD patients. We also compared MNA score with anthropometric measurement to ensure its effectiveness in MHD patients.

## Materials and methods

After approval from the hospital ethical review committee (ERC Reference No. 116-NEPH-022021), we conducted a prospective cross-sectional study at The Kidney Centre Postgraduate Training Institute Karachi, Pakistan (TKC-PGTI). We included adult ESRD patients on MHD three times a week with each session lasting for four hours at our center. Patients with speech and cognitive impairment were excluded from the study.

After getting written informed consent, demographic data (age, gender, occupation, presence of diabetes mellitus, and dialysis details like duration, frequency) was collected on a preformed proforma. One of the co-primary investigators (Co-PI) did anthropometric measurements (height, weight, BMI), mid-arm circumference (MAC), calf circumference (CC), and tricuspid skin fold thickness (TSFT) and asked questions from MNA, at the bedside before the hemodialysis session. BMI was calculated by dividing body weight by the square of height (Kg/m^2^). MAC and CC were measured using a measuring tape. TSFT was measured using caliper. The mid-arm muscle circumference (MAMC) was calculated using the following formula: MAMC = MAC (cm)-π TSFT (mm)/10. The BMI and MAMC were evaluated with the reference values given by the World Health Organization (WHO). The normal range of BMI is 18.5 to 24.5 Kg/m^2^, of MAMC in male=25.3 mm, female=23.2 mm and TSFT in male=12.5, female=16.5 mm respectively [14]. A MAMC of less than 90% indicates protein depletion; greater than 90% indicates adequate or ample protein reserves [15].

Mini nutritional assessment (MNA) contains 18 items and it evaluates four different aspects; anthropometric measurements (BMI, weight loss, mid-arm and mid-calf circumferences); general assessment (lifestyle, medications, mobility and signs of depression); short dietary assessment (number of meals, food and fluid intake) and subjective assessment (self-perception of food and nutrition). Patients were divided into three groups according to the score. Scores less than 17 out of 30 are considered malnourished, 17-23.5 are at the risk of malnutrition, 24 and above are considered normal [10].

Statistical Analyses were performed by using IBM SPSS version 21.0 (IBM Corp., Armonk, NY). Continuous variables were expressed in mean ± STD, while frequencies and percentages were obtained for categorical variables. Chi-square or Fisher’s exact test was applied to see any associations between variables. A p-value of less than or equal to 0.05 was considered significant.

## Results

We enrolled 195 patients in our study of which 127 (65.1%) were males and 68 (34.9%) were females. Among our study group, most women were stay-at-home mothers (57, 29.2% overall and 83.8% among all women), while most men owned their own businesses (44, 22.6%). The mean age was 51.2±14 years and the mean duration of hemodialysis was 4.6±4.1 years. Seventy-five (38.5%) had diabetes mellitus. Most of our patients belonged to the middle socioeconomic group (110, 56.6%) (Table 1).

**Table 1:** Baseline characteristics of patients (n=195)

Baseline Characteristics		n (%)
Socioeconomic status	Lower	35 (17.9)
	Middle	110 (56.5)
	Upper	50 (25.6)
Occupation	Un-employed	35 (17.9)
	Stay-home mother	57 (29.2)
	Retired	37 (19)
	Businessman	44 (22.6)
	Employed	22 (11.3)

Table 2 shows the frequency of response to subjective assessment and malnutrition status as per MNA. Most of our patients live independently (192, 98.5%) and take more than three drugs/day (190, 97.4%). Out of 195 patients, 13 (6.7%) had acute psychological stress in the past three months. We found out that most of our study population were at risk of developing malnutrition (112, 57.4%); however, only 9 (4.6%) patients were malnourished.

**Table 2:** Frequency of response and malnutrition status as per MNA

Response and nutritional assessment according to mini nutritional assessment (MNA)		n (%)
Food intake decreased over the last three months	No	160 (82.1)
	Moderate	33 (16.9)
	Severe	2 (1)
Weight loss in last three months	No	153 (78.5)
	1-3 kg	37 (19)
	> 3 kg	5 (2.6)
Mobility	Fully active	134 (68.7)
	Some limitations	60 (30.8)
	Bed or chair bound	1 (0.5)
Neuropsychological disease	No	150 (76.9)
	Mild dementia	44 (22.6)
	Severe dementia or depression	1 (0.5)
Number of meal /day	2	45 (23.1)
	3	150 (76.9)
Selected consumption of protein intake	No	136 (69.7)
	2 servings	52 (26.7)
	3 servings	7 (3.6)
Fluid/day	< 3 cups	35 (17.9)
	3-5 cups	84 (43.1)
	>5 cups	76 (39)
Mode of feeding	Self-feed without difficulty	173 (88.7)
	Self-feed with difficulty	18 (9.2)
	Unable to feed by himself	4 (2.1)
Self-view of nutritional status	No problem	102 (52.3)
	Uncertain	66 (33.8)
	Views self as malnourished	27 (13.8)
Self-view as compared to people of same age	Not as good as others	50 (25.6)
	As good as others	114 (58.5)
	As better than others	31 (15.9)
Total assessment by using MNA	Normal (24-30 points)	74 (37.9)
	At risk of malnutrition (17-23.5 points)	112 (57.4)
	Malnourished (< 17 points)	9 (4.6)

Anthropometric measurements are illustrated in Table 3. 

**Table 3:** Anthropometric measurements

Anthropometric measurements		n (%)
Body mass index (BMI)	< 19	31 (15.9)
	19-20.9	16 (8.2)
	21-22.9	43 (22.1)
	≥ 23	105 (53.8)
Mid-arm circumference (MAC) in cm	< 21	2 (1)
	21-21.9	5 (2.6)
	≥ 22	188 (96.40)
Calf circumference (CC) in cm	< 31	116 (59.50)
	≥ 31	79 (40.5)

In the study group, gender was not associated with MNA score (p=0.839), while age was significantly associated with MNA (p=0.004). We observed that most malnourished patients belonged to the age group of >65 years (5, 56.6%). BMI was also significantly associated with MNA (p<0.001). Among all malnourished patients, the patients with BMI of <19 were 5 (55.6%), on the other hand, most of the patients who were normal according to MNA, belonged to a BMI group of ≥23 (52, 70.3%). Similarly, MAMC, MAC, and CC were also significantly associated with MNA (p<0.05). Diabetes mellitus, social, and other demographic factors were not associated with the nutritional status of our patients (Table 4).

**Table 4:** Subgroup analysis of study population according to MNA and anthropometric measurements Values are given in terms of n (%)

Variables		Malnourished 74 (37.9)	At the risk of malnourishment 112 (57.4)	Normal 9 (4.6)	p-value
Gender	Male	6 (66.7)	71 (63.4)	50 (67.6)	0.839
	Female	3 (33.3)	41(36.6)	24 (32.4)	
Age	≤ 35 years	1 (11.1)	22 (19.6)	14 (18.9)	0.004
	36-50 years	2 (22.2)	24 (41.1)	26 (35.1)	
	51-65 years	1 (11.1)	46 (41.1)	29 (39.2)	
	> 65 years	5 (55.6)	20 (17.9)	5 (6.5)	
Occupation	No job	2 (22.2)	20 (17.9)	13 (17.6)	0.202
	House wife	1 (11.1)	35 (23.2)	21 (28.4)	
	Retired	3 (33.3)	26 (23.2)	8 (10.8)	
	Business	3 (33.3)	21(18.8)	20 (27)	
	On job	0	10 (8.9)	12 (16.2)	
Diabetes mellitus	Yes	2 (22.2)	48 (42.9)	25 (33.8)	0.272
	No	7 (77.8)	64 (57.1)	49 (66.2)	
Lives independently	Yes	9 (100)	110 (98.2)	73 (98.6)	0.999
	No	0	2(1.8)	1(1.4)	
Socioeconomic status	Lower	1 (11.1)	21(18.8)	13 (17.6)	0.973
	Middle	5 (55.6)	63 (56.3)	42 (56.8)	
	Upper	3 (33.3)	28 (25)	19 (25.7)	
Diabetes mellitus	Yes	2 (22.2)	48 (42.9)	25 (33.8)	0.272
	No	7 (77.8)	64 (57.1)	49 (66.2)	
Body mass index (BMI)	< 19	5 (55.6)	23 (20.5)	3 (4.1)	<0.001
	19-20.9	1 (11.1)	12 (10.7)	3 (4.1)	
	21-22.9	1 (11.1)	26 (23.2)	16 (21.6)	
	≥ 23	2 (22.2)	51 (45.5)	52 (70.3)	
Mid-arm muscle circumference (MAMC)	< 90	6 (66.9)	43 (38.4)	18 (24.3)	0.016
	≥ 90	3 (33.3)	69 (61.6)	56 (75.7)	
Mid-arm circumference (MAC)	< 21	0	2 (1.8)	0	0.036
	21 - 22.9	2 (22.2)	2 (1.8)	1 (1.4)	
	≥ 22	7 (77.8)	108 (96.4)	73 (98.6)	
Calf circumference (CC)	< 31	7 (77.8)	78 (69.6)	31 (41.9)	<0.001
	≥ 31	2 (22.2)	34 (30.4)	43 (58.1)	

## Discussion

Many nutritional assessment tools are used in the general population, but most require trained personnel or specific equipment. Anthropometric measurements require training for the use of calipers. Operator dependent variability is a possibility. Most of these tools are less reliable for comparing nutritional status and mortality in MHD patients and some like BMI shows reverse epidemiological correlation as compared to the general population [16]. Previously we did a study of nutritional assessment in which we did bedside anthropometric measurements only [13]. In this study, we evaluated nutritional status of MHD patients using MNA and compared it with anthropometric measurements. MNA has been designed to provide a rapid assessment of nutritional status in elderly patients in outpatient clinics, hospitals, and nursing homes [17]. It showed similar efficacy as a diagnostic tool of malnutrition compared to SGA, which is more time-consuming and requires trained staff [18].

Most of our study patients were male and young, which comprises the working class in our country. This is comparable to other studies [19]. Age is an important factor, as the number of elderly MHD patients are increasing globally. MNA was used initially as a nutritional assessment tool for elderly with normal renal function. Early detection of PEW in elderly MHD patients can be crucial. Soysal et al. evaluated frailty status by Fried’s criteria (unintentional weight loss, exhaustion, low levels of activity, weakness, and slowness) and MNA, found that frailty status is associated with the nutritional status, and MNA showed a strong correlation with Fried’s frailty criteria in older adults [20]. The majority of our patients at risk of malnutrition belonged to the age group 51-65 years (46, 41.1%) and more than 65 years (20, 17.9%).

When we evaluated the response to different questions of MNA, we found that most of our study patients were taking three meals a day (150, 76.9%) and felt as good as other people of the same age group (114, 58.5%). Most of our patients are fully active (134, 68.7%). This could be due to strict adherence to MHD protocols and regularity of hemodialysis.

In our study, we found a strong association of MNA with BMI (p <0.001). Most of our patients had a normal BMI (105, 52.8%), but the majority were at risk of malnutrition (112, 57.4%). These results are similar to another study from Pakistan [21]. Similar to BMI, anthropometric measurements showed a strong association with MNA (MAC, p=0.036; CC, p<0.001; MAMC, p=0.016). As compared to our previous study [13], MNA showed comparative effectiveness to anthropometric measurements alone in this study. Occupation, socioeconomic status and presence of diabetes mellitus did not show any association with MNA in our study.

Limitations

Our study has a few limitations. It is a single-center study, we have not inquired about dietary routine of patients, and we have not compared MNA with other nutritional assessment tools like SGA. We compared MNA with anthropometric measurements alone and found comparative efficacy in MHD patients. Our study is done in one of the biggest hemodialysis units in Pakistan. We found MNA as a simple, easy to use tool for nutritional assessment in MHD patients. This study will help nephrologists and renal nutritionists for better and timely management of MHD patients, as nutritional status correlates well with morbidity and mortality.

## Conclusions

Chronic kidney disease (CKD) is a growing problem globally involving all age groups, including the working class. Despite being done at one specialist nephrology center, our study highlighted the importance of nutritional status and its assessment by using MNA, which is an easy and simple bedside scoring system. Timely and correct assessment of nutritional status helps renal nutritionists and nephrologists for the proper treatment of malnutrition.
